# Gonadotropin-Releasing Hormone in Regulation of Thymic Development in Rats: Profile of Thymic Cytokines

**DOI:** 10.3390/ijms20164033

**Published:** 2019-08-19

**Authors:** Victoria I. Melnikova, Nadezhda V. Lifantseva, Svetlana N. Voronova, Liudmila A. Zakharova

**Affiliations:** Koltsov Institute of Developmental Biology, Russian Academy of Sciences, 119334 Moscow, Russia

**Keywords:** rat fetus, thymic development, gonadotropin-releasing hormone, GnRH receptor, thymic cytokines, prenatal programming

## Abstract

An increasing body of recent experimental data confirms the impact of neurohormones on fetal development and function of different body systems. The synthesis of many neurohormones starts in fetal tissues before the hypothalamic–pituitary–adrenal and hypothalamic–pituitary–gonadal systems are formed, and their high levels are detected in the bloodstream. Here, we studied the role of gonadotropin-releasing hormone (GnRH) in rat thymus development and tried to reveal possible mechanisms underlying the GnRH effects in early development. Western blotting and reverse transcription-polymerase chain reaction allowed us to identify receptor for GnRH in the fetal thymus with peak expression on embryonic days 17–18 (ED17–18). Blocking the receptors in utero on ED17 by a GnRH antagonist suppressed the concanavalin A-induced proliferative response of T cells in adults. GnRH (10^−7^ M) increased mRNA expression of interleukin (IL)-4, IL-10, IL-1β, interferon γ (IFNγ), and tumor necrosis factor α (TNFα) in the thymus of 18-day fetuses after an ex vivo culture for 24 h. The increased mRNA levels of the cytokines in the thymus were accompanied by increased numbers of CD4+ T helpers. Overall, the data obtained confirm the regulatory or morphogenetic effect of GnRH on fetal thymus development mediated by synthesis of thymic cytokines.

## 1. Introduction

Gonadotropin-releasing hormone 1 (GnRH) is the primary regulator of the reproductive system, responsible for pituitary gonadotropin release, which eventually regulates the secretion of sex steroids [1,2]. In addition to the hypothalamic–pituitary–gonadal (HPG) system, GnRH is an important component of immune system modulation. The immunomodulatory effects of GnRH include the prevention of thymic atrophy, stimulation of T-cell proliferation, activation of natural killer cells, and modulation of cytokine production [2,3]. In adults, GnRH and its receptor are expressed in the thymus, spleen, and peripheral blood lymphocytes [4,5,6]. GnRH can be produced and secreted by T lymphocytes either spontaneously or after induction by external stimuli [7]. These data suggest that GnRH may be involved in the regulation of immune functions in an autocrine or paracrine manner. However, GnRH concentration is low in the bloodstream in adults. In contrast, GnRH concentration in plasma on embryonic day 18 (ED18) in rat fetuses enormously exceeds those in postnatal life. The forebrain is considered to be the principal source of GnRH in general circulation before the establishment of the blood–brain barrier in fetal rats [8,9]. The amounts of brain-derived GnRH delivered to the bloodstream likely suffice to influence the potential peripheral targets in fetal rats. It has been demonstrated that GnRH contributes to the regulation of cell-mediated immunity during intrauterine development in rats. The treatment of fetuses in utero with either GnRH receptor antagonist (GnRH-ant) or anti-GnRH antibodies resulted in the suppression of concanavalin A (Con A)-induced proliferative responses of fetal thymocytes determined 48 h later [9]. However, it remains unclear how long the GnRH effects are maintained. The important role of GnRH and sex steroids in bidirectional programming of both neuroendocrine and immune function was clearly demonstrated during early postnatal development in rats [10]. The blockade of central and peripheral GnRH receptors within five days after birth resulted in the suppression of cell-mediated and humoral immune responses up to the age of three months and altered the proportions between different thymocyte subclasses. This was accompanied by complete blockade of luteinizing hormone secretion at around 15 days of age, altered pituitary GnRH receptor expression, and the absence of gonadotropin priming at the sex accessory organ level [10]. Therefore, the immunomodulatory effects of GnRH during postnatal development are most probably mediated via sex steroids.

This study aimed to determine the role of GnRH in the regulation of thymic development before the establishment of the HPG axis. The ontogenetic pattern of the GnRH receptor expression in the fetal thymus was determined. GnRH-ant was administered to fetuses in utero on ED17 when the GnRH receptor expression peaked, and the development of cell-mediated immune response in the thymus was evaluated on postnatal days 20 and 40 (PND20 and PND40). Furthermore, the influence of GnRH on the cytokine expression in the fetal thymus ex vivo was investigated.

## 2. Results

### 2.1. GnRH Receptor Expression in the Developing Thymus

**Figure 1:**
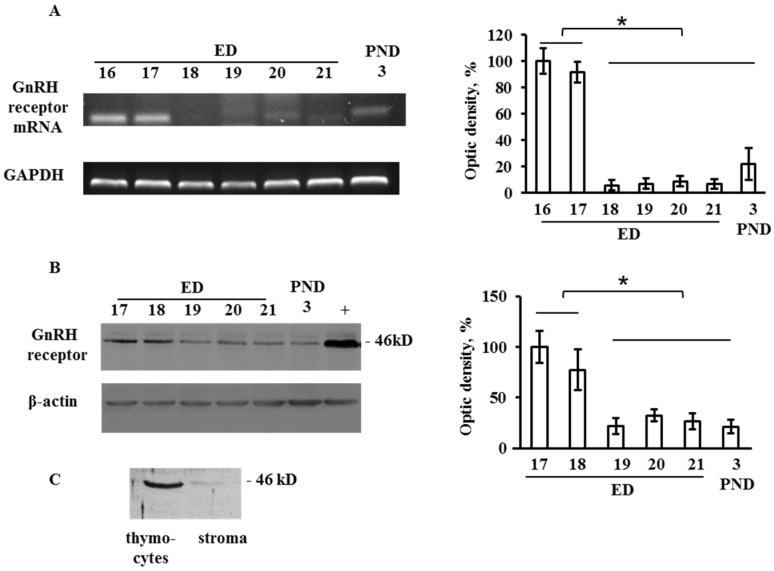
Ontogenetic pattern of the gonadotropin-releasing hormone (GnRH) receptor expression in the developing thymus. (**A**) mRNA of GnRH receptor revealed by RT-PCR in the rat thymus on embryonic days (ED) 16, 17, 18, 19, 21 and postnatal day 3 (PND3). (**B**) Western blot assay of GnRH receptor in the rat thymus on ED17, ED18, ED19, ED20, ED21, and PND3. The anterior pituitary (PND3) was used as a positive control (+). (**C**) Protein expression of GnRH receptor in thymocytes and thymic stromal elements on ED18. Plots represent the optic density of the corresponding bands. Bars indicate the means ± SEM of three independent experiments; * *p* < 0.05 using one-way ANOVA test.

Both mRNA and protein of GnRH receptor were detected in the fetal thymus at all studied developmental stages from ED16 to PND3. The maximum mRNA level was observed on ED16 and ED17 (Figure 1A), while the protein level of GnRH receptor peaked on ED17–ED18 (Figure 1B). GnRH receptor was detected in thymocytes but not in thymic stromal cells (Figure 1C). A faint band on the blot is most likely due to insignificant contamination of the stroma by thymocytes since the ideal separation of cells is unfeasible.

### 2.2. Long-Term Effects of GnGH Receptor Blockade in Rat Fetuses 

**Figure 2:**
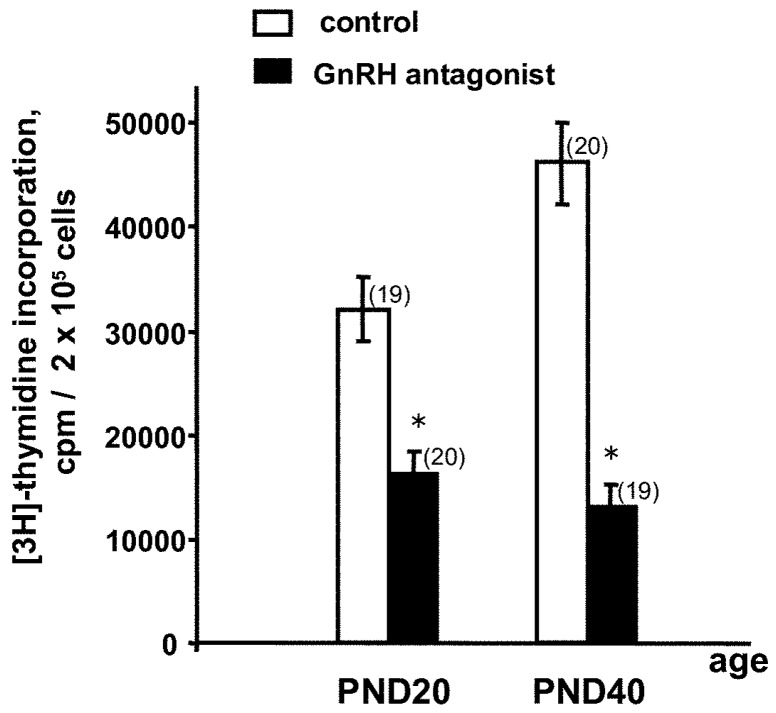
Concanavalin A (Con A)-induced (2.5 µg/mL) proliferative response of lymphocytes from the rat thymus on postnatal days PND20 and PND40 after a single in utero administration of GnRH antagonist to the fetuses (2 µg in 20 mL 0.9% NaCl per fetus) on ED17. Control fetuses were administered the same saline volume. Bars indicate the means ± SEM of four independent experiments; in total, the litters from eight pregnant rats were analyzed (9–10 rats per litter). For each experiment two pregnant rats were used (1-control and 1-GnRH-ant). One half of the litter from each pregnant rat was analyzed on PND20, and the other half on PND40. The numbers of rats in each experimental group are indicated in brackets; * *p* < 0.05 vs. control using the Mann–Whitney U-Test.

A single administration of GnRH-ant to the fetuses on ED17 significantly decreased the ConA-induced proliferative response of T cells on PND20 and PND40 (Figure 2). In contrast, a single administration of GnRH-ant to rat pups on PND3 did not alter their proliferative activity on PND20 (31,870 + 2740 cpm in control and 29,486 + 3184 cpm in GnRH-ant injected rats). 

### 2.3. GnRH Influence on T Lymphocyte Differentiation in Organotypic Culture of Fetal Thymus

**Figure 3:**
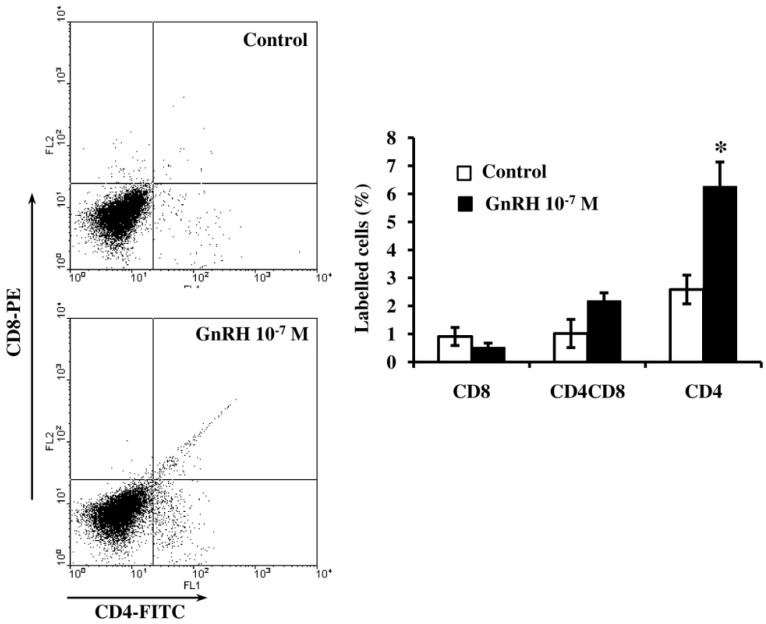
Effect of GnRH (10^−7^ M) on the differentiation of T cells in an organotypic culture of thymi from ED18 fetuses after five days in vitro. Flow cytometry analysis of CD4+, CD8+ and CD4CD8+ cells. Bars represent the percentage of labelled cells ± SEM of three independent experiments; * *p* < 0.05 vs. control using the Mann–Whitney U-Test.

Culturing ED18-thymocytes with GnRH (10^−7^ M) for five days doubled the proportion of CD4+ T cells (Figure 3). At the same time, a trend to an increased proportion of double-positive T cells was observed.

### 2.4. GnRH Influence on Synthesis and Secretion of Cytokines in Fetal Thymus

**Figure 4:**
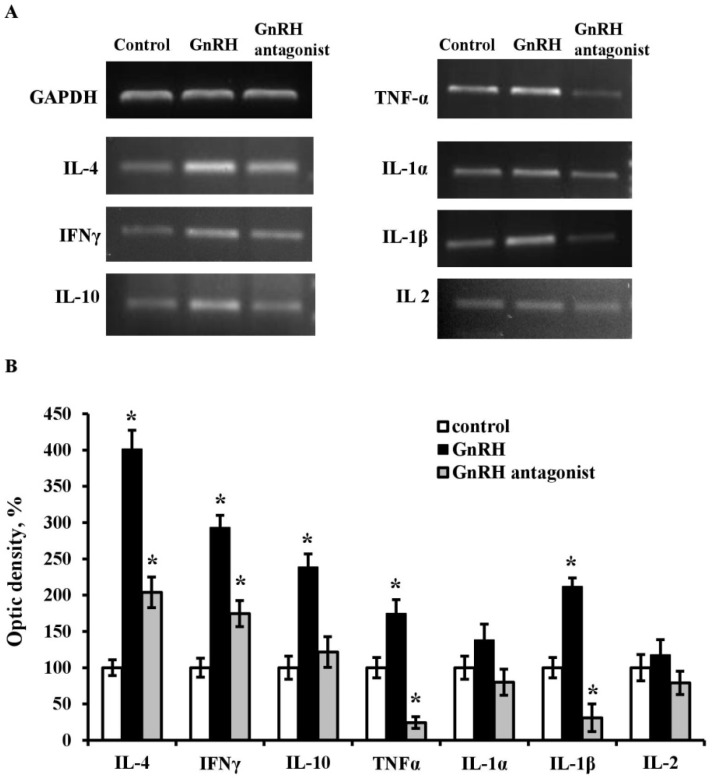
Effect of GnRH (10^−7^ M) and GnRH-antagonist (10^−7^ M) on the cytokine mRNA expression revealed by RT-PCR in the fetal thymus on ED18 after an ex vivo culture for 24 h. (**A**) PCR products; (**B**) relative expression levels of cytokines (the optic density of the bands). Bars indicate the means ± SEM of three independent experiments; * *p* < 0.05 vs. control using the Mann–Whitney U-Test.

Culturing ED18-thymi with GnRH (10^−7^ M) for 24 h increased mRNA levels of nearly all studied cytokines except interleukin (IL)-2 and IL-1α, whose expression remained unaltered for one day. The most pronounced mRNA expression changes were observed for IL-4, IL-10, IL-1β, interferon γ (IFNγ), and tumor necrosis factor α (TNFα) (Figure 4). GnRH-ant (10^−7^ M) suppressed IL-1β and TNFα, enhanced IL-4 and IFNγ mRNA expression compared to control, while the mRNA levels of IL-1α, IL-2 and IL-10 remained unaltered.

**Figure 5:**
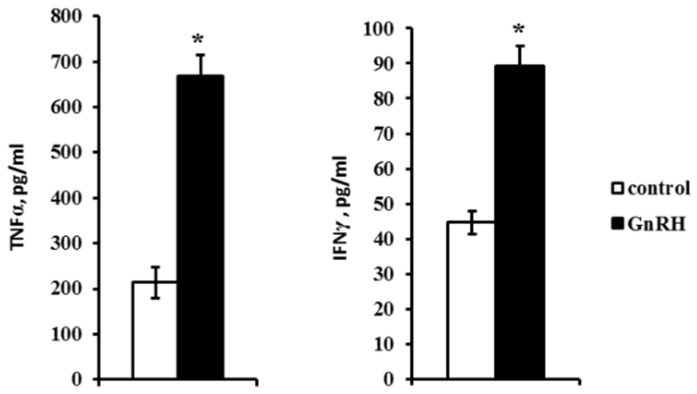
Effect of GnRH (10^−7^ M) on the secretion of interferon γ (IFNγ) and tumor necrosis factor α (TNFα) by thymocytes on ED18 after an ex vivo culture for 24 h. The detection limit for rat cytokines was 40 pg/mL. Bars indicate the means ± SEM of three independent experiments; * *p* < 0.05 vs. control using the Mann–Whitney U-Test.

The quantitation of cytokines in the incubation medium demonstrated that the levels of IL-2, IL-4 and IL-1α were lower than 40 pg/mL, which is below the method sensitivity. The level of IFNγ was 40 pg/mL in control but increased twice in the presence of GnRH (Figure 5). The initially high level of TNFα (about 200 pg/mL) also increased almost threefold after the exposure to GnRH (Figure 5). At the same time, it had no effect on the secretion of IL-10, which remained steady at 300 pg/mL.

## 3. Discussion

The above experiments aimed at elucidating the role of the neuropeptide GnRH in the development of thymic T cells in the rat fetuses. We focused on the remote effect of prenatal GnRH receptor blockade by GnRH-ant [9] on the function of T cells and tried to reveal possible mechanisms underlying this hormone functions, in particular those affecting the synthesis and secretion of regulatory thymic cytokines. The age-related expression pattern of the GnRH receptor, which was detected in the adult thymus [5], was studied. The blockade of central and peripheral GnRH receptors in utero by GnRH-ant at the peak of their transcription, on ED17, suppressed the Con A-induced proliferative response of T cells in adults (Figure 1 and Figure 2). The suppression of the T cell proliferative response has been demonstrated previously in the fetuses after in utero administration of GnRH-ant, while GnRH canceled this dysfunction [9,11].

Our data indicate that, in contrast to the prenatal period, a single GnRH-ant administration to rat pups on PND3 had no effect on the functional activity of T cells until PND20. At the same time, Morale et al. [10] demonstrated that the chronic antagonist administration to rats during the neonatal period suppressed both T and B immunity five days later. After a neonatal exposure to GnRH-ant, adult rats and primates demonstrated decreased thymic weight and numbers of mature T and B cells in the lymphoid tissues and peripheral blood [10,12]. Different GnRH effects on the immune system can be mediated by different regulatory mechanisms. In rat fetuses, the hypothalamic control of the pituitary secretion of gonadotropins is established by ED21 alongside with the formation of axonal pathways of GnRH transport to the portal circulation and the onset of the expression of GnRH receptor in gonadotropes, which is most pronounced on PND10–12 [13,14]. In this context, the revealed GnRH-ant effects are most likely due to its direct impact on the thymus on ED17. The high expression level of GnRH receptor on ED17–18 as well as the absence of remote effects of a single GnRH-ant administration in the neonatal period indicate the effect of GnRH on thymic development most likely in the late second prenatal decade. The GnRH effects following chronic exposure to the antagonist in the neonatal period are most likely mediated by pituitary gonadotropins [10].

The administration of GnRH and its agonists normalizes the numbers of lymphocytes, predominantly, CD4+ T helpers, as well as their functional activity in adults and prevents age-related thymic involution [15,16,17]. In our experiments, GnRH doubled the number of CD4+ T cells in the embryonic thymus in ex vivo model (Figure 3). 

Although the expression of GnRH receptor was detected only in thymocytes (Figure 1), the effect of GnRH on the differentiation of thymic stromal elements cannot be excluded. The embryonic thymus is a dynamic system where the stromal elements and thymocytes actively interact, and differentiation of thymic stromal elements depends on their interaction with the primary lymphopoietic precursors [18] populating the epithelial primordium of the thymus on ED13–16 [19].

The mechanisms underlying the GnRH effect on the immune system remain underexplored and depend on many factors such as the hormone dose and exposure time, gestation period, sex, age, species, and metabolic peculiarities. For instance, it was shown that the lymphocyte sensitivity to GnRH differs in the thymus and spleen. After neonatal administration of GnRH-ant, the mitogenic proliferative response is completely blocked in thymocytes and partially suppressed in more mature spleen lymphocytes [10]. Possible mechanisms controlling lymphocyte numbers in rats include the GnRH capacity to induce the expression of IL-2 receptor γ in T cells, and thus regulate the IL-2-dependent Con A-induced proliferative response [20,21]. However, this mechanism can be realized in the postnatal period. Our data indicate that physiological concentrations of GnRH induce no IL-2 synthesis in an organotypic culture of ED18-thymuses. At the same time, GnRH significantly increases the synthesis of IL-4, IL-10, TNFα, and IFNγ (Figure 4). GnRH has a most pronounced impact on the synthesis of the lymphocyte differentiation factor IL-4 which is known to regulate the synthesis of other cytokines in synergy with IL-10. The synthesis of some cytokines is suppressed by GnRH-ant in fetal thymus after an ex vivo culturing. IL-1β and TNFα mRNA expression decrease about 4.0-fold compared to control. The mRNA levels of IL-1α, IL-2 and IL-10 remain unaltered, while IL-4 and IFNγ mRNA expression slightly increase. These data indicate that synthesis of cytokines are under GnRH regulation in rat thymus during intrauterine development.

Two stages of the synthesis of cytokines, which control lymphocyte proliferation and differentiation, are recognized in the mouse fetal thymus [22]. mRNA of IL-1β, IL-4, IL-5, IL-6, IL-7, IFNγ, and TNFβ are detectable from ED14 to ED20; and that of IL-1α, IL-2, and IL-3 from ED16. At the same time, the synthesis of IL-7 stimulating T cell proliferation but not differentiation diminishes by the time of IL-2 synthesis, which indicates the involvement of certain cytokines in certain stages of thymic development. Cytokines are known to function in the adult mammalian thymus as a “minor cytokine network” composed of short-range factors that mediate cell interactions and induce the synthesis of one another [23]. A similar function can be expected for cytokines in early thymic development. In our experiments, the GnRH-induced increase in the levels of cytokine mRNAs was accompanied by the increased number of CD4+ T helpers where many of these cytokines are synthesized.

The effect of GnRH on the secretion of thymic cytokines was not uniform. The concentration of the regulatory cytokine IL-10, whose mRNA level was relatively high, remained unaltered after the exposure to GnRH. The levels of IL-2, IL-4 and IL-1α were below the method sensitivity (40 pg/mL) and undetectable in the culture medium. At the same time, GnRH doubled and tripled the secretion of pleiotropic cytokines IFNγ and TNFα, respectively (Figure 5). These cytokines are known to contribute to the regulation of cell-to-cell interactions, apoptosis, and morphogenesis [24]. TNFα level in peripheral blood has not been assayed in different animal and human models, but its high level (more than 250 pg/mL) has been revealed in inflammation and septic shock [25,26]. In our experiments, GnRH induced both the synthesis and secretion of TNFα. Its content in the organotypic thymic culture was 650 pg/mL, which can be attributed to the absence of the neuroendocrine control. Analysis of the obtained data suggests that the GnRH can impact on the thymic development through cytokine network.

The possible effect of the neuroendocrine system on thymic development is indirectly confirmed by previous data on the suppressive effect of GnRH-ant on Con A-induced proliferative activity of thymocytes in an in vivo rat fetus model. The suppressive effect is not observed after the exposure of fetal thymocytes to the antagonist in culture. A twofold decrease in the GnRH level observed in the fetal thymus after hypothalamectomy supports this assumption. Administration of GnRH to the fetuses immediately after hypothalamectomy restores the Con A-induced proliferative response of thymocytes, while the same treatment of sham-operated fetuses has no effect on their immune response [9].

In addition, the GnRH level in the thymus can be modulated by sex steroids, in particular, testosterone [4], whose synthesis by Leydig cells becomes detectable in the rat fetus starting from ED18.5–19.5 [27]. Receptors for androgens and estrogens in the thymus are expressed as early as during embryonic development, with their level increasing by birth [28]. Injection of testosterone, estrogen, or their derivatives to chick or quail embryos results in atrophy of the bursa of Fabricius, degeneration of lymphoid tissue in follicles and its substitution by fibrous tissue, and disturbances in the development of thymic stromal elements creating the microenvironment for lymphocyte maturation [29]. A drop in the level of sex hormones after pre- or post-pubertal castration of male rats results in increasing cellularity and hypertrophy of the thymus efficiency. According to Azad et al. [4], in castrated two-month male rats, the level of GnRH in the thymus increases, whereas the concentration of its precursor decreasing, but substitution therapy with testosterone prevents these effects. The authors suppose that one of factors responsible for these processes is a rise in the level of GnRH, which stimulates lymphocyte proliferation in the thymus. Sex steroids modulate the molecular processing of GnRH precursor and, consequently, the levels of GnRH in tissues. The concentration of GnRH mRNA in castrated animals remains unchanged, which is evidence that testosterone has a post-translational effect, inhibiting GnRH precursor processing into GnRH itself [4].

In addition to the neuroendocrine regulation, thymic development is also influenced by GnRH synthesized directly in the fetal thymus starting from ED18 [9]. The regulation of thymic development is likely realized by two different GnRH forms considering that GnRH1 is synthesized in the hypothalamus, while GnRH2 is largely synthesized outside of the brain. The extracerebral GnRH2 synthesis was detected in the ovary, testis, prostate, and mammary gland plus maternal placenta during pregnancy [6,30]. Thus, arguably, the effects of circulating GnRH can be realized by endocrine mechanisms; while those of the locally synthesized GnRH, by autocrine/paracrine mechanisms. 

## 4. Materials and Methods

### 4.1. Animals and Experimental Design

Pregnant Wistar rats (Stolbovaya Breeding Center, Moscow, Russia) weighing 250–300 g and fetuses were used in this study (the day of conception was designated as ED1). Animals were kept in standardized conditions (24 °C, 12:12 h light–dark cycle, food and water ad libitum).

The ontogenetic pattern of GnRH receptor expression in the thymus was analyzed by Western blotting and reverse transcription-polymerase chain reaction (RT-PCR) in rats from ED16 to PND3. The intracellular distribution of the receptor was investigated through the analysis of the GnRH receptor protein expression on ED18 [31] separately for thymocytes and thymic stromal cells. Freshly isolated thymi were homogenized in a glass homogenizer and filtered through a Falcon™ cell strainer with 40 µm nylon mesh. Thymocytes were harvested by centrifugation of the obtained suspension, while thymic stroma samples were collected from the mesh surface. The obtained samples were used for Western blotting.

The functionality of GnRH receptor as well as possible mechanisms of GnRH effects in the developing thymus were analyzed by evaluating cytokine synthesis and secretion in an organotypic culture of thymi from ED18 fetuses. 

To evaluate long-term effects of prenatal blockade of central and peripheral GnRH receptors on thymus development, pregnant rats on the 17th day of gestation were anesthetized with pentobarbital (50 mg/kg body), and the fetuses in utero were injected intraperitoneally with the selective GnRH-ant [D-pGlu-D-Phe-D-Trp-Ser-Tyr-D-Trp-Leu-Arg-Pro-Gly-NH2] (Sigma, St. Louis, Mo, USA) at the dose of 2 µg per fetus in 20 mL of 0.9% NaCl. Fetuses from control pregnant rats were injected with an equal volume of saline. Con A-induced proliferative response of thymocytes was analyzed on PND20 and PND40.

In special experiments, GnRH-ant was administered to neonatal rats (PND3, 50 µg/rat, intraperitoneally), whereas control animals received equal saline volume. Con A-induced proliferative response of thymocytes was analyzed on PND20. 

All manipulations with animals were performed in accordance with the European Convention on the Protection of Vertebrate Animals Used for Experimental and Other Scientific Purposes (Strasburg, 1986) and approved by the Ethics Committee for Animal Research of the Koltzov Institute of Developmental Biology (Russian Academy of Sciences, approval code: 20, approved on 18 January 2018).

### 4.2. Cell Preparation and Con A-Induced Proliferation Assay

The thymi were gently homogenized in 1 mL of RPMI-1640 (Sigma, St. Louis, Mo, USA). The suspension was passed through a nylon mesh and washed twice with RPMI-1640 by centrifugation (400× *g* for 10 min). The cell viability estimated by trypan blue exclusion was about 95%. Con A-induced proliferative response of thymocytes was assessed as described previously [9]. Thymocytes (2.5 × 10^5^ cells/mL) were plated at 200 µL/well in 96 well plates with Con A (2.5 µg/mL, Sigma, St. Louis, Mo, USA). Cultures were incubated at 37 °C, 95:5 air/CO_2_, in a humidified incubator for 72 h. [^3^H]-thymidine (0.5 µCi/well, Amersham, Amersham, UK) was added for the last 18 h of culture. Cells were harvested onto GF-C glass fiber filters (Whatmann, Little Chalfont, UK) and [^3^H]-thymidine incorporation was measured in a β-scintillation counter (LKB, Stockholm, Sweden). Each experimental point was repeated five or six times.

### 4.3. Western Blot Analysis of GnRH Receptor Expression in Thymus

Western blotting was performed as described previously [31]. The thymi were homogenized at 4 °C in RIPA buffer (150 mM NaCl, 1.0% NP40, 0.5% sodium deoxycholate, 0.1% SDS, and 50 mM Tris-HCl, pH 8.0) with Protease Inhibitor Cocktail Set III (Merk, Darmstadt, Germany) and centrifuged (12,000× *g* for 20 min at 4 °C). The supernatants (cleared homogenates) were used for further investigation. Protein concentration was measured using a BCA Protein Assay Kit (The Thermo Scientific Pierce, New York, NY, USA) according to the manufacturer’s instruction. 

SDS-PAGE in 12% gel (10 µL of cleared homogenates, 40 µg of total protein per lane) was performed according to Laemmli [32]. Separated proteins were transferred to a nitrocellulose membrane in transfer buffer (25 mM Tris-HCl, pH 7.5, 192 mM glycine, 20% ethanol) and blots were incubated overnight at 4 °C with antibodies to GnRH receptor (1:1000, Alomone Labs, Jerusalem, Israel) or actin (1:10,000, Sigma, St. Louis, Mo, USA). Immunoreactive bands were visualized after incubation with peroxidase-conjugated secondary antibodies (1:10,000, Jackson Immunoresearch, West Grove, Pa, USA) using the ECL detection system (Amersham Biosciences, Amersham, UK) and X-ray film. The image was analyzed using the ImageJ software (https://imagej.nih.gov/ij/index.html). The relative quantities (optical densities) of the immunoreactive bands on X-ray film were measured as the gray level (GL) which is related to the optical density (OD) of the specimen as follows [33]: ODSpecimen − ODBackground = log(GLBackground) − log(GLSpecimen). The dependence of the optical density on the amount of the protein subjected to Western blotting was evaluated preliminarily. For the further procedure, the protein amount was chosen within the linear range of detection.

### 4.4. Evaluation of GnRH Influence on the Numbers of CD4+, CD8+, and CD4+CD8+ T Cells in Fetal Thymus Organotypic Culture

The organotypic culture was prepared according to the protocol described by Cunningham with co-authors [34]. Thymi from ED18-fetuses were cultured for 5 days in RPMI-1640 (Sigma, St. Louis, MO, USA) containing 10% fetal calf serum at 1 mL/well in 18-well plates (6 thymi per well) in the presence of 10^−7^ M GnRH. Culture was maintained at 37 °C, 95:5 air/CO_2_, in a humidified incubator. Culture medium was changed daily. On the 5th day, thymocytes were isolated as described above and incubated for 1 h with antibodies to CD4 antigen conjugated with fluorescein isothiocyanate (FITC) and to CD8 antigen conjugated with phycoerythrin (PE) (1:20, Cederlain, Burlington, Ontario, Canada). Then cells were washed twice and analyzed on a FACSCalibur flow cytometer (BD Biosciences, Franklin Lakes, NJ, USA). 

### 4.5. Evaluation of GnRH Influence on the Synthesis and Secretion of Cytokines

Thymi from ED18-fetuses were cultured for 24 h in RPMI-1640 (Sigma, St. Louis, Mo, USA) in the presence of 10^−7^ M GnRH or 10^−7^ M GnRH-ant (6 thymi per well, in 1 mL of medium). Cytokine mRNAs were detected by RT-PCR as described below and the cytokine culture medium levels were determined by cytometric bead array (CBA).

### 4.6. Cytokine Assay by Cytometric Bead Array

IFNγ, IL-1α, IL-1β, IL-2, IL-4, IL-10, and TNFα were determined by CBA according to the manufacturer’s protocol (BD Biosciences, Franklin Lakes, NJ, USA). Samples (50 μL) were analyzed in duplicate using a CBA kit on a FACSCalibur cytometer (BD Biosciences, Franklin Lakes, NJ, USA). The cytokine levels were quantified using the CellQuestPro and CBA Software (BD Biosciences, Franklin Lakes, NJ, USA). The detection limit for rat cytokines was 40 pg/mL.

### 4.7. RNA Extraction and RT-PCR Analysis

Total RNA was isolated using TRIzol^®^ (Invitrogen, Thermo Fisher Scientific Inc., New York, NY, USA) according to the manufacturer’s instructions. Contaminating DNA in RNA preparations was digested by incubation with DNase (ThermoFisher Scientific, New York, NY, USA) for 15 min at 22 °C. The reaction was stopped by 1 µL EDTA (25 mM) and heating at 65 °C. Total RNA was precipitated by ethanol; RNA pellets were dissolved in water and denatured by incubation at 65 °C for 15 min. The total RNA concentration was measured using a NanoDrop 8000 spectrophotometer (Thermo Scientific, New York, NY, USA).

RNA (2 µg) was reverse transcribed by M-MuLV Reverse Transcriptase (New England Biolabs, Ipswich, UK) according to the manufacturer’s protocol. The reaction was terminated by heating at 95° C for 5 min. The presence of genomic DNA contamination in the RNA preparations was checked by reverse transcriptase negative controls (no reverse transcriptase in the reaction) in half of each RNA sample. 

PCR amplification was carried out using Colored-Taq polymerase (Silex M, Moscow, Russia). cDNA (1 µL) was amplified in 25 µL PCR mix for 35 cycles. Cross-contamination was checked using water instead of cDNA in the reaction mixture. Detection of glyceraldehyde-3-phosphate dehydrogenase (GAPDH) transcript using GAPDH primers served as a control for RNA integrity and the RT-PCR process. The PCR products were subjected to electrophoresis on a 2% agarose gel and visualized by ethidium bromide staining. A DNA ladder (Invitrogen, Thermo Fisher Scientific Inc., USA) was used to determine the size of the PCR products. The intensity of different bands in PCR gels was visualized and quantified using the ChemiDoc MP Imaging System and Image Lab Touch Software (Biorad, Hercules, Ca, USA). Primers were designed using the Primer3 software (Whitehead/MIT Center for Genome Research, Cambridge, UK). Primer sequences are presented in Table 1.

### 4.8. Statistical Analysis

Results are expressed as mean ± standard error of the mean (SEM) from at least three independent experiments. Statistical analysis was conducted using Sigma Stat 3.5 software (San Jose, CA, USA). The Mann–Whitney U-Test was used to compare independent groups. One-way ANOVA for non-parametric data was utilized to compare more than one group with each other. The difference was considered to be statistically significant at *p* < 0.05.

## 5. Conclusions

The data obtained in this work point to the significance of GnRH in thymic development. A prenatal blockade of GnRH receptor disturbs the thymic developmental program and later the function of T cells. During early development, the effect of GnRH is mediated, probably, by cytokines whose synthesis and secretion in the thymus is upregulated by the hormone.

**Figure 6:**
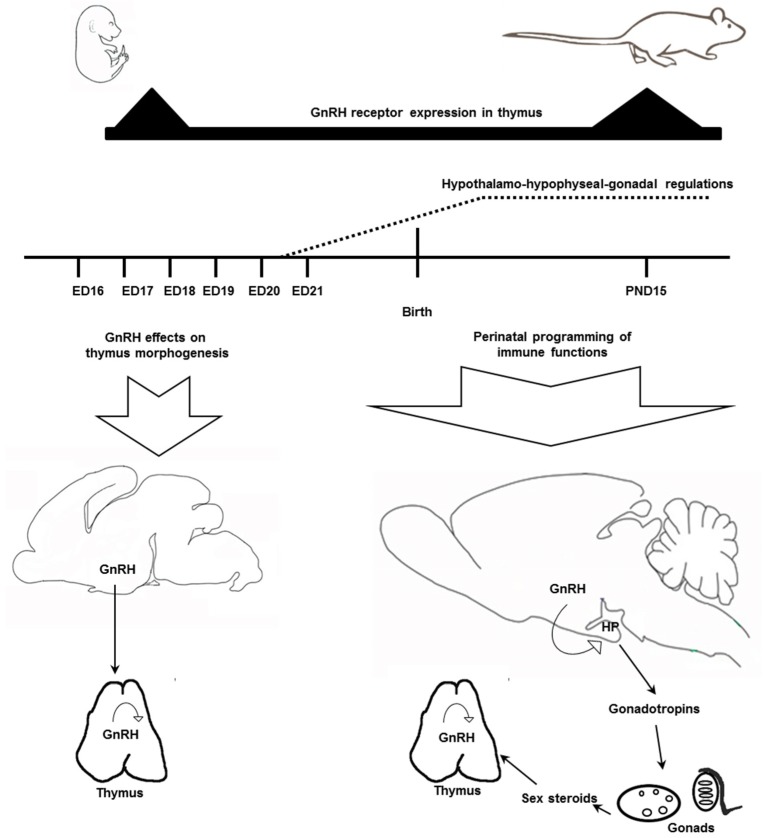
Schematic representation of GnRH effects on the thymus development in fetal and early postnatal rats. During prenatal development up to ED20–21, before the establishment of the HPG endocrine regulations, hypothalamic GnRH can be released to general circulation and provide a direct effect on the morphogenesis of thymus. Since the development of blood–brain barrier and the establishment of the HPG axis in early postnatal life GnRH is involved in the bidirectional programming of both neuroendocrine and immune functions via gonadotropins and sex steroids. In addition to the neuroendocrine regulation, the effects of GnRH synthesized in thymus can be realized by autocrine/paracrine mechanisms. The developmental pattern of the GnRH receptor expression in the thymus is in favor of this assumption. ED—embryonic day, PND—postnatal day, HP—hypophysis.

Analysis of the published and obtained data suggests that the GnRH impact on the immune system varies during ontogeny. In early development, neurohormones including GnRH control growth and differentiation of tissues that belong to different body systems including the immune system [35,36,37]. In prenatal development up to ED20–21, before the establishment of the HPG endocrine regulations, GnRH can provide a direct long-term effect on the morphogenesis of thymus (Figure 6). During the perinatal period GnRH is involved in the programming of the immune functions via the neuroendocrine axis.

It is early development when the epigenetic mechanisms providing for the adaptive plasticity of the immune system are realized. In adults, many hormones including GnRH participate in the immune response control in addition to their specific functions. Alterations in their physiological concentrations during this period induce short-term changes in the development of lymphoid precursors of T and B cells, which consequently modulates the immune function.

## Figures and Tables

**Table 1:** Polymerase chain reaction (PCR) primers.

Gene	Forward Primer	Reverse Primer	Product Size (bp)
GnRHR	caggacccacgcaaactacag	tgtatatggacaaggctgctaacc	403
IL-2	ccctgcaaaggaaacacagc	caaatccaacacacgctgca	202
IL-10	tgctcttactggctggagtg	cctggggcatcacttctacc	264
IFN-γ	ccctctctggctgttactgc	cgaacttggcgatgctcatg	315
IL-1α	cccagatcagcacctcacag	gcgagtgacttaggacgagg	570
IL-1β	tcaagcagagcacagacctg	ttctgtcgacaatgctgcct	357
IL-4	ctcatctgcagggcttccag	agtgttgtgagcgtggactc	173
TNF-α	ccatgagcacggaaagcatg	ggctcataccagggcttgag	586
GAPDH	tacaacctccttgcagctcc	ggatcttcagaggtagtctgtc	378

## Abbreviations

CBA	cytometric bead array
Con A	concanavalin A
ED	embryonic day
GAPDH	glyceraldehyde-3-phosphate dehydrogenase
GnRH	gonadotropin-releasing hormone
GnRH-ant	GnRH receptor antagonist
HPG	hypothalamic-pituitary-gonadal
IFNγ	interferon γ
IL	interleukin
PND	postnatal day
RT-PCR	reverse transcription-polymerase chain reaction
TNFα	tumor necrosis factor α

## References

[1] Tomaszewska-Zaremba D., Herman A. (2009). The role of immunological system in the regulation of gonadoliberin and gonadotropin secretion. Reprod. Biol..

[2] Zakharova L.A., Izvolskaia M.S., Kahn S.M. (2012). Interactions between the reproductive and immune systems during ontogenesis: The role of GnRH, sex steroids and immunomediators. Sex steroids.

[3] Quintanar J.L., Guzmán-Soto I. (2013). Hypothalamic neurohormones and immune responses. Front. Integr. Neurosc..

[4] Azad N., LaPaglia N., Agrawal L., Steiner J., Uddin S., Williams D.W., Lawrence A.M., Emanuele N.V. (1998). The role of gonadectomy and testosterone replacement on thymic luteinizing hormone-releasing hormone production. J. Endocrinol..

[5] Jacobson J.D., Crofford L.J., Sun L., Wilder R.L. (1998). Cyclical expression of GnRH and GnRH receptor mRNA in lymphoid organs. Neuroendocrinology.

[6] Tanriverdi F., Silveira L.F.G., MacColl G.S., Bouloux P.M.G. (2003). The hypothalamic–pituitary–gonadal axis: Immune function and autoimmunity. J. Endocrinol..

[7] Levite M. (2008). Neurotransmitters activate T-cells and elicit crucial functions via neurotransmitter receptors. Curr. Opin. Pharmacol..

[8] Ugrumov M.V., Sapronova A.Y., Melnikova V.I., Proshlyakova E.V., Adamskaya E.I., Lavrentieva A.V., Nasirova D.I., Babichev V.N. (2005). Brain is an important source of GnRH in general circulation in the rat during prenatal and early postnatal ontogenesis. Comp. Biochem. Physiol. A Mol. Integr. Physiol..

[9] Zakharova L.A., Ermilova I.Y., Melnikova V.I., Malyukova I.V., Adamskaya E.I. (2005). Hypothalamic control of the cell-mediated immunity and of the Luteinizing Hormone-Releasing Hormone level in thymus and peripheral blood of rat fetuses. Neuroimmunomodulation.

[10] Morale M.C., Batticane N., Bartoloni G., Guarcello V., Farinella Z., Galasso M.G., Marchetti B. (1991). Blocade of central and peripheral luteinizing hormone-releasing hormone (LHRH) receptors in neonatal rats with a potent LHRH-antagonist inhibits the morphofunctional development of the thymus and maturation of the cell-mediated and humoral immune responses. Endocrinology.

[11] Zakharova L.A., Malyukova I.V., Proshlyakova E.V., Sapronova A.Y., Ugrumov M.V. (2000). Hypothalamo-pituitary control of the cell-mediated immunity in rat embryos: Role of LHRH in regulation of lymphocyte proliferation. J. Reprod. Immunol..

[12] Mann D.R., Akinbami M.A., Lunn S.F., Fraser H.M., Gould K.G., Ansari A.A. (2000). Endocrine-immune interaction: Alteractions in immune function resulting from neonatal treatment with a GnRH antagonist and seasonality in male primates. Am. J. Reprod. Immunol..

[13] Huhtaniemi I. (1995). Molecular aspects of the ontogeny of the pituitary-gonadal axis. Reprod. Fertil. Dev..

[14] Dygalo N.N., Shemenkova T.V., Kalinina T.S., Shishkina G.T. (2014). A critical point of male gonad development: Neuroendocrine correlates of accelerated testicular growth in rats during early life. PLoS ONE.

[15] Marchetti B., Guarcello V., Morale M.C., Bartolini G., Raiti F., Palumbo G., Farinella Z., Cordaro S., Scapagnini U. (1989). LHRH agonist restoration of age associated decline of thymus weight, thymic LHRH receptors and thymocyte proliferative capacity. Endocrinology.

[16] Jacobson J.D., Ansari M.A., Mansfield M.E., McArthur C.P., Clement L.T. (1999). Gonadotropin-releasing hormone increases CD4 T-lymphocyte numbers in an animal model of immunodeficiency. J. Allergy Clin. Immunol..

[17] Ullewar M.P., Umathe S.N. (2014). Gonadotropin-releasing hormone agonist selectively augments thymopoiesis and prevents cell apoptosis in LPS induced thymic atrophy model independent of gonadal steroids. Int. Immunopharmacol..

[18] Brelinska R., Malinska A. (2005). Homing of hemopoietic precursor cells to the fetal rat thymus: Intercellular contact-controlled cell migration and development of the thymic microenvironment. Cell Tissue Res..

[19] Anderson G., Jenkinson W.E., Jones T., Parnell S.M., Kinsella F.A., White A.J., Pongrac’z J.E., Rossi S.W., Jenkinson E.J. (2006). Establishment and functioning of intrathymic microenvironments. Immunol. Rev..

[20] Batticane N., Morale M.C., Gallo F., Farinella Z., Marchetti B. (1991). Luteinizing hormone-releasing hormone signaling at the lymphocyte involves stimulation of interleukin-2 receptor expression. Endocrinology.

[21] Tanriverdi F., Gonzalez-Martinez D., Hu Y., Kelestimur F.P., Bouloux M.G. (2005). GnRH-I and GnRH-II have differential modulatory effects on human peripheral blood mononuclear cell proliferation and interleukin-2receptor g -chain mRNA expression in healthy males. Clin. Exp. Immunol..

[22] Montgomery R.A., Dallman M.J. (1991). Analysis of cytokine gene expression during fetal thymic ontogeny using the polymerase chain reaction. J. Immunol..

[23] Yarilin A.A., Belyakov I.M. (2004). Cytokines in the thymus: Production and biological effects. Cur. Med. Chem..

[24] Beutler B.A. (1999). The role of tumor necrosis factor in health and disease. J. Rheumatol. Suppl..

[25] Kothari N., Bogra J., Abbas H., Kohli M., Malik A., Kothari D., Srivastav S., Singh P.K. (2013). Tumor necrosis factor gene polymorphism results in high TNF level in sepsis and septic shock. Cytokine.

[26] Sharova V.S., Izvolskaia M.S., Zakharova L.A. (2015). Lipopolysaccharide-induced maternal inflammation affects the GnRH neuron development in fetal mice. Neuroimmunomodulation.

[27] Rouiller-Fabre V., Levacher C., Pairault C., Racine C., Moreau E., Olaso R., Livera G., Migrenne S., Delbes G., Habert R. (2003). Development of the foetal and neonatal testis. Andrologia.

[28] Staples J.E., Gasiewicz T.A., Fiore N.C. (1999). Estrogen receptor alpha is necessary in thymic development and estradiol-induced thymic alterations. J. Immunol..

[29] Razia S., Maegawa Y., Tamotsu S., Oishi T. (2006). Histological changes in immune and endocrine organs of quail embryos: Exposure to estrogen and nonylphenol. Ecotoxicol. Environ. Saf..

[30] Ramakrishnappa N., Rajamahendran R., Lin Y.M., Leung P.C. (2005). GnRH in non-hypothalamic reproductive tissues. Anim. Reprod. Sci..

[31] Melnikova V.I., Sharova N.P., Maslova E.V., Voronova S.N., Zakharova L.A. (2010). Ontogenesis of rat immune system: Proteasome expression in different cell populations of the developing thymus. Cell. Immunol..

[32] Laemmli U.K. (1970). Cleavage of structural proteins during the assembly of the head of bacteriophage T4. Nature.

[33] Smolen A.J., Conn P.M. (1990). Image analytic techniques for quantification of immunohistochemical staining in the nervous system. Methods in Neurosciences, Quantitative and Qualitative Microscopy.

[34] Cunningham C.A., Teixeiro E., Daniels M.A., Bosselut R.S., Vacchio M. (2016). FTOC-Based Analysis of Negative Selection in T-Cell Development. Methods in Molecular Biology.

[35] Morgan H.D., Santos F., Green K., Dean W., Reik W. (2005). Epigenetic reprogramming in mammals. Hum. Mol. Genet..

[36] Zakharova L.A. (2009). Plasticity of neuroendocrine-immune interactions during ontogeny: Role of perinatal programming in pathogenesis of inflammation and stress-related diseases in adults. Recent Pat. Endocr. Metab. Immune Drug Discov..

[37] Wu X.Q., Li X.F., Ye B., Popat N., Milligan S.R., Lightman S.L., O’Byrne K.T. (2011). Neonatal programming by immunological challenge: Effects on ovarian function in the adult rat. Reproduction.

